# Next-generation sequencing identified novel Desmoplakin frame-shift variant in patients with Arrhythmogenic cardiomyopathy

**DOI:** 10.1186/s12872-020-01369-5

**Published:** 2020-02-11

**Authors:** Xiaoping Lin, Yuankun Ma, Zhejun Cai, Qiyuan Wang, Lihua Wang, Zhaoxia Huo, Dan Hu, Jian’an Wang, Meixiang Xiang

**Affiliations:** 1 Department of Cardiology, the Second Affiliated Hospital, Zhejiang University School of Medicine, 88 Jiefang Road, Hangzhou, 310009 Zhejiang China; 2 Department of Radiology, the Second Affiliated Hospital, Zhejiang University School of Medicine, 88 Jiefang Road, Hangzhou, Hangzhou, 310009 Zhejiang China; 3 Experimental Teaching Center, School of Basic Medical Sciences, Zhejiang University, 866 Yuhangtang Road, Hangzhou, 310058 Zhejiang China; 4 Department of Cardiology and Cardiovascular Research Institute, Renmin Hospital of Wuhan University, 238 Jiefang Road, Wuhan, 430060 China; 5 Provincial Key Lab of Cardiovascular Research, 88 Jiefang Road, Hangzhou, 310009 Zhejiang China

**Keywords:** Arrhythmogenic cardiomyopathy, Next generation sequencing, Genetic variant, Desmoplakin

## Abstract

**Background:**

Arrhythmogenic cardiomyopathy (AC) is one of the leading causes for sudden cardiac death (SCD). Recent studies have identified mutations in cardiac desmosomes as key players in the pathogenesis of AC. However, the specific etiology in individual families remains largely unknown.

**Methods:**

A 4-generation family presenting with syncope, lethal ventricular arrhythmia and SCD was recruited. Targeted next generation sequencing (NGS) was performed and validated by Sanger sequencing. Plasmids containing the mutation and wild type (WT) were constructed. Real-time PCR, western-blot and immunofluorescence were performed to detect the functional change due to the mutation.

**Results:**

The proband, a 56-year-old female, presented with recurrent palpitations and syncope. An ICD was implanted due to her family history of SCD/ aborted SCD. NGS revealed a novel heterozygous frame-shift variant (*c.832delG*) in Desmoplakin (*DSP*) among 5 family members. The variant led to frame-shift and premature termination, producing a truncated protein. Cardiac magnetic resonance (CMR) of the family members carrying the same variant shown myocardium thinning and fatty infiltration in the right ventricular, positive bi-ventricular late gadolinium enhancement and severe RV dysfunction, fulfilling the diagnostic criteria of AC. HEK293T cells transfected with mutant plasmids expressed truncated *DSP* mRNA and protein, upregulation of nuclear junction plakoglobin (*JUP*) and downregulation of β-catenin, when compared with WT.

**Conclusion:**

We infer that the novel *c.832delG* variant in *DSP* was associated with AC in this family, likely through Wnt/β-catenin signaling pathway.

## Background

Arrhythmogenic cardiomyopathy (AC), characterized by gradual myocardium loss and fibrofatty replacement predominately in the right ventricle [1], is one of the primary causes for life-threatening ventricular arrhythmia and sudden cardiac death (SCD), particularly in young and athletes [2]. The clinical presentations vary, including palpitations, syncope, symptomatic ventricular tachycardia, right heart failure and SCD. Sometimes, SCD was the only manifestation in AC patients, posting tremendous challenges to the diagnosis post mortem [2, 3]. Diagnosis of AC, according to the guideline proposed by the international task force [4], is mainly based on findings of electrophysiological, structural and histological features, family history and genetic testing, hence, for those SCD patients, their family screening is of utmost importance. The current treatments for AC are mostly supportive and palliative [5], aiming at alleviation of arrhythmic and heart failure symptom and prevention of SCD, and heart transplantation is the final solution for end-stage patients. However, reversal or a complete cure of the disease requires further in-depth understanding of its etiology and pathogenesis.

Known as genetically determined cardiomyopathy, AC is mainly inherited in an autosomal dominant pattern with genetic and phenotypic heterogeneity [6]. Genetic studies have identified mutations in 5 components of cardiac desmosomes as main etiology of AC [6], namely Plakophilin 2 (*PKP2*), Desmoplakin (*DSP*), Desmoglein 2 (*DSG2*), Desmocollin 2 (*DSC2*), and Junction plakoglobin (*JUP*). Genetic defects of above genes can be found in 40–60% of AC patients [4]. However, the specific etiology in individual case remains largely unknown. First identified in a recessive disorder of keratoderma, woolly hair, and AC with left ventricle predominance (Carvajal syndrome) [7], *DSP* mutations are responsible for nearly 2–12% of AC patients [8, 9]. Recent study interestingly found that the left ventricle predominance or bi-ventricle involved phenotypes were associated with *DSP* non-missense mutations [10], but the genotype-phenotype correlations remain uncertain due to small sample size and need to be further characterized in individual families as well as large sample cohorts. Recent studies also suggested mutations that impaired ion channel activities may be causal or modifier to AC [11, 12], however, their prevalence is unsure.

In the current study, the underlying genetic defects in a 4-generation family presenting syncope, life-threatening ventricular arrhythmia and SCD were explored using next generation high-throughput sequencing platform, and a novel frame-shift variant *c.832delG* in *DSP* was identified. Cardiac magnetic resonance (CMR) further reveled the diagnosis of AC on two asymptomatic family members carrying the identical *DSP* variant. Through co-segregation and genotype-phenotype association analysis, and functional study on HEK293T cells, we infer that the novel frame-shift variant *DSP c.832delG* was associated with AC in this family.

## Methods

### Study subjects

The study protocol conforms to the ethical guidelines of the 1975 Declaration of Helsinki and was approved by Institutional Review Board (IRB) at the Second Affiliated Hospital, Zhejiang University School of Medicine (2016–087). Written informed consent was obtained from all participants. Ten out of total 31 family members in a 4-generation SCD family were recruited in the current study. A complete clinical information including family history, medical history, physical examination, lab test, 12-lead echocardiogram (ECG), 24-h Holter monitoring, transthoracic echocardiography and CMR were collected.

### DNA extraction, target region capture and next-generation sequencing

The proband was selected for next generation sequencing using a commercial capture array (Roche NimbleGen, WI, USA) covering the exons and 50 base pairs of adjacent introns of 1876 cardiovascular diseases associated genes, including inherited cardiomyopathy, arrhythmogenic diseases, congenital heart diseases, mitochondrial diseases, etc. 

Genomic DNA was extracted from peripheral blood lymphocytes by standard procedures using Axygen® AxyPrep™-96 Blood Genomic DNA Kit (Axygen, NY, United States). The DNA libraries were constructed and sequenced using the Illumina 2000 platform (Illumina, CA, United States), providing an average sequencing depth of > 100-fold of targeted exons.

### Data filtering and bioinformatics analysis

The screening algorithms for potential disease-causing variants were as follows. Initially, intronic and synonymous exonic variants were excluded. Secondly, matched population and in-house database minor allele frequencies (MAF) were used to rule out common variants, defined by MAF > 0.01. MAF of 3 major SNP databases were compared: ExAc (http://exac.broadinstitute.org/), 1000 genomes (http://www.1000genomes.org/) and ESP6500 (http://evs.gs.washington.edu/EVS/). Thirdly, rare non- synonymous variants were examined with HGMD (http://www.hgmd.cf.ac.uk/ac/), OMIM (http://www.omim.org/) and ClinVar databases (https://www.ncbi.nlm.nih.gov/clinvar/) and finally analyzed using 3 known prediction tools, namely PolyPhen-2 (http://genetics.bwh.harvard.edu/pph2/), SIFT (http://sift.jcvi.org/) and MutationTaster (http://www.mutationtaster.org/), and categorized according to the recommended guidelines of the American College of Medical Genetics and Genomics (ACMG) and the Association for Molecular Pathology [13]. Sanger sequencing was performed bidirectionally for the verification of *AKAP9 c.10714C > G*, *FLNC c.7778C > G*, *SYNE1 c.25954C > T* and *DSP c.832delG* in all participants.

### Plasmids construction and site-directed mutagenesis

AICSDP-9:DSP-mEGFP was a gift from the Allen Institute for Cell Science (Addgene plasmid # 87424; http://n2t.net/addgene:87424; RRID:Addgene_87,424) [14]. In order to facilitate the observation following transfection of mutant plasmid, GFP were cleaved and inserted in between the promoter and *DSP* gene. The frame-shift mutation was introduced into a wild-type *DSP* clone using a QuikChange II XL Site-Directed Mutagenesis Kit (Stratagene, La Jolla, CA, USA). The clones were sequenced to confirm the desired mutation and to exclude any other sequence variations.

### RT-PCR and real-time PCR

HEK293T cells were transfected with either blank, wild type or mutant plasmids using lipofectamine 3000 (Invitrogen, MA, USA) according to the manufacturer’s instructions. Total RNA was extracted from transfected cells using the Trizol reagent (Invitrogen, MA, USA). cDNA was synthesized using PrimeScript RT reagent Kit (Takara, Shiga, Japan). The resulting cDNA was subjected to real-time PCR using TB Green Premix Ex Taq kits (Takara, Shiga, Japan) on an Applied Biosystems 7500 Fast Real-Time PCR System (ABI, CA, USA). The primers named “N-terminal” detected the mRNA levels in the N-terminal side of the *DSP* mutation site, and the primers named “C-terminal” detected the mRNA levels in the C-terminal side of the *DSP* mutation site. GAPDH was used as an endogenous control.

The sequences of primers were listed as follows:

N-terminal-F: 5′-GCAGGATGTACTATTCTCGGC-3′,

N-terminal-R: 5′-CCTGGATGGTGTTCTGGTTCT-3′;

C-terminal-F: 5′-ACATCATTCAGGCCACGT-3′;

C-terminal-R: 5′- CCAGTTGACTCATGCGTA-3′;

GAPDH-F: 5′-CGCTCTCTGCTCCTCCTGTT-3′;

GAPDH-R: 5′-CCATGGTGTCTGAGCGATGT-3′.

### Western blots

24 h after transfection, total cell extracts were lysed by RIPA lysis buffer. Nuclear and cytoplasmic extracts were separated using Nuclear and Cytoplasmic Protein Extraction Kit (Beyotime Biotechnology, Shanghai, China). Next, proteins were separated by sodium dodecyl sulfate poly-acrylamide gel electrophoresis (SDS-PAGE) and transferred to polyvinylidene fuoride (PVDF) membranes. The membranes were blocked for 1 h in a blocking solution of 5% (w/v) non-fat milk in PBS containing 0.1% (v/v) Tween-20 and incubated at 4 °C overnight with indicated primary antibodies. Primary antibodies included antibodies against *JUP* (1:1000, sc-8415, Santa Cruz Biotechnology, CA, USA), β-catenin (1:1000, ab6302, Abcam, Cambridge, UK), GFP (1:1000, AF1483, Beyotime Biotechnology), GAPDH (1:5000, 3683S, Cell Signaling Technology, MA, USA), Lamin B1 (1:1000, ET1606–27, HuaBio antibodies, China). Excess primary antibodies were washed off, and then the membranes were incubated with secondary antibodies conjugated with horseradish peroxidase for 1 h at room temperature. The western blot bands were visualized were visualized using the enhanced chemiluminescence western blotting detection system (Bio-Rad, CA, USA).

### Immunofluorescence analysis

Cells seeded on cover slips were fixed with 4% paraformaldehyde (PFA)/PBS, permeabilized in 0.5%(v/v) Triton X-100 (Sigma-Aldrich, MO, USA) and blocked with 5% (w/v) BSA. Then the cells were incubated using the antibody mouse-anti-JUP (1:1000, sc-8415, Santa Cruz Biotechnology) overnight at 4 °C, followed by secondary antibodies anti-mouse Alexa Fluor 594 (1:200, Thermo Fisher, A-21203, CA, USA) incubation in 5% BSA in PBS for 1 h at room temperature. Finally, coverslips were mounted on microscope slides using mounting medium contained with DAPI (H-1200, Vector, CA, USA). Images were acquired using a fluorescence microscope (Leica, IL, USA). Colocalization analysis between *JUP* and nuclear was performed by Coloc 2 ImageJ in random high-power fields. Pearson’s correlation coefficient was used to represent the colocalization quantification, + 1 for perfect correlation, 0 for no correlation, and − 1 for perfect anti-correlation. Optical confocal microscopies of cells were obtained using Leica TCS SP8 (Leica Microsystems Inc).

### Statistical analysis

Data were presented as the means ± SEM of at least three independent experiments. Student T test was performed to evaluate differences of continuous variables between two groups. One-way ANOVA was used for comparison among three groups. *P* values of less than 0.05 were considered statistically significant. Statistical calculations were carried out using GraphPad Prism 8.0.1.

## Results

### Demographic and clinical features of family members

**Fig. 1:**
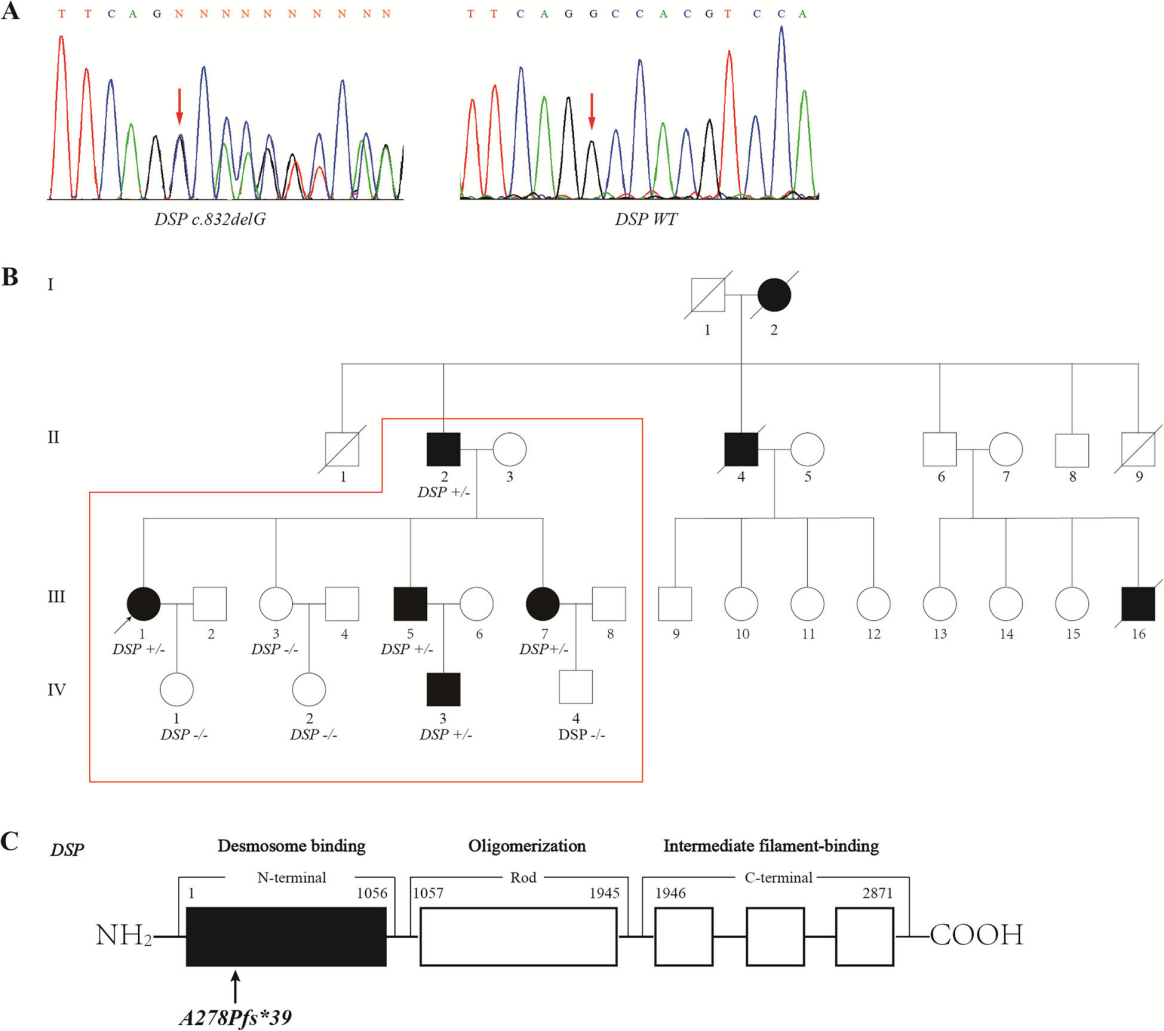
Genetic analysis and in silico prediction. **a** A heterozygous frame-shift variant *DSP c.832delG* was identified through targeted next generation sequencing; **b** Pedigree and genotype. Family members in the red frame were genotyped. Arrow indicates the proband; squares indicate male family members; circles indicate female members; black filled indicate family members diagnosed with AC or experienced sudden cardiac death; diagonal lines indicate deceased family member; **c** Schematic diagram of the location of DSP p.A278Pfs*39

The pedigree of the family was shown in Fig. 1b. The proband (III-1), a 56-year-old female, was admitted to our hospital due to ICD battery depletion. She presented with a history of recurrent palpitations and syncope for 10 years. An ICD was implanted when she was 49 years old due to a positive family history of SCD/aborted SCD. Since no discharge was detected upon ICD implantation and she remained asymptomatic, no medication was administrated. Her paternal grandmother (I-2), uncle (II-4), and cousin (III-16) died suddenly. Her youngest sister (III-7) experienced 2 episodes of syncope in her 38 and 40 years old, and an ICD was implanted in her 40 years old following resuscitation from a VT/VF event. Six appropriate discharges were detected in the following 6 years, and a second ICD was replaced when she was 46 years old. She was generally asymptomatic with β-blocker. Ten out of 31 family members were available and recruited for subsequent clinical and genetic evaluations (Fig. 1b).

**Fig. 2:**
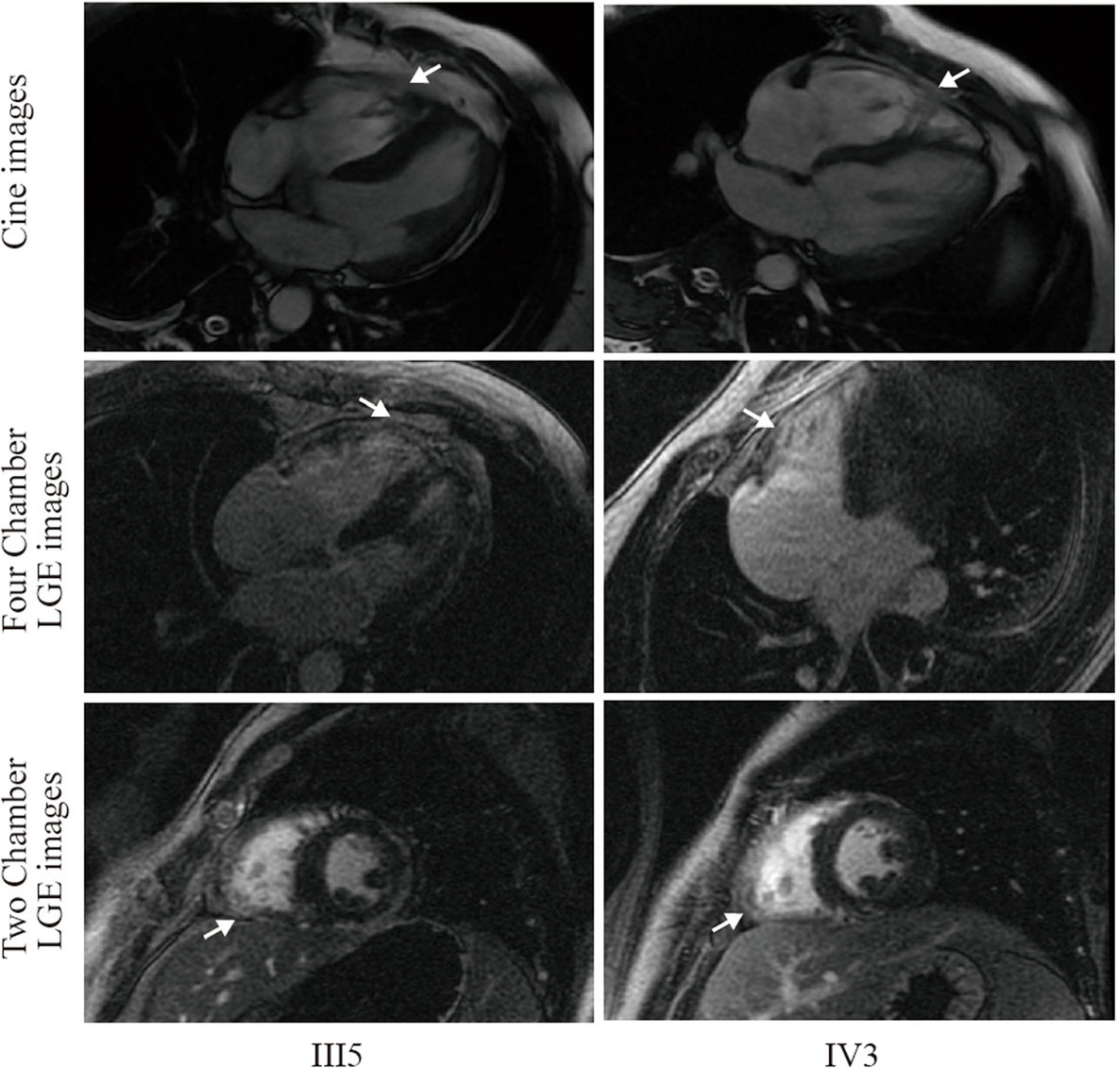
Representative cardiac magnetic resonance images. Myocardium thinning and fatty infiltration (arrow) in the right ventricular and positive bi-ventricular late gadolinium enhancement were detected in III5 and IV3. Myocardium thinning and fatty infiltration (arrow) were detected in the right apical region in III-3. LGE, late gadolinium enhancement

The complete clinical features of all available family members were summarized in Table 1. No obvious depolarization and repolarization or structural abnormalities were detected by either ECG or transthoracic echocardiography tests for all participants. Though III-3, III-5 and IV-3 were asymptomatic, CMR were performed due to their potential positive genotype. Myocardium thinning and fatty infiltration was detected in the right apical area in III-3 when cardiac function was preserved. However, other than myocardium thinning and fatty infiltration in the right ventricle, positive bi-ventricular late gadolinium enhancement (LGE) and sever right ventricular dysfunction were detected in III-5 and IV-3. In addition, left ventricular function was moderately affected in IV-3 (Table 1 and Fig. 2). Thus, CMR manifestation of III-5 and IV-3 fulfilled the international Task Force criteria for the diagnosis of AC [4].

**Table 1:** Clinical featuresand genotypes of family members

No.	Gender	Age rangs (y)	Medical history	ECG	Holter	ECHO	CMR	AC Criteria	Genotype
II2	M	80–85	CHD, Dual-chamber pacemaker	DDD pacing rhythm, LBBB, old MI	DDD pacing rhythm	LA enlargement	–	*–*	*AKAP9+/−FLNC−/−DSP+/−*
II3	F	80–85	HTN	PVCs	Frequent PVCs, PACs	Normal	–	*–*	*AKAP9−/−FLNC+/−DSP−/−*
III1	F	55–60	Palpitation, syncope, ICD	Normal	Frequent PVCs, NSVT	Normal	–	Fulfilled	*AKAP9+/−FLNC+/−DSP+/−*
III3	F	50–55	Palpitation	PVCs	–	–	RV apical myocardium thinning and fatty infiltration	Unfulfilled	*AKAP9+/−FLNC−/−DSP−/−*
III5	M	50–55	Asymptomatic	Normal	–	Normal	RV myocardium thinningand myocardial fatty infiltration, bi-ventricular LGE, LVEF52%, RVEF29%	Fulfilled	*AKAP9−/−FLNC−/−DSP+/−*
III7	F	45–50	Syncope, VT/VF, ICD	Low QRS voltages	Frequent PVCs	Normal	–	Fulfilled	*AKAP9+/−FLNC+/−DSP+/−*
IV1	F	30–35	Asymptomatic	Normal	–	Normal	–	*–*	*AKAP9+/−FLNC−/−DSP−/−*
IV2	F	25–30	Asymptomatic	PVCs	–	–	–	*–*	*AKAP9−/−FLNC−/−DSP−/−*
IV3	M	20–25	Asymptomatic	PVCs	–	Normal	RV myocardium thinningand myocardial fatty infiltration, bi-ventricular LGE, LVEF43%, RVEF17%	Fulfilled	*AKAP9−/−FLNC−/−DSP+/−*
IV4	M	15–20	Asymptomatic	Normal	–	–	–	*–*	*AKAP9−/−FLNC+/−DSP−/−*

*ECG* electrocardiogram; *ECHO* echocardiogram; *CMR* cardiac magnetic resonance; *F* female; *M* male; *CHD* coronary artery disease; *HTN* hypertension; *LBBB* left bundle branch block; *MI* myocardial infarction; *LA* left atrium; *LV* left ventricle; *RV* right ventricle; *EF* ejection fraction; *PACs* premature atrial contractions; *PVCs* premature ventricular contractions; *NSVT* non-sustained ventricular tachycardia; *VT* ventricular tachycardia; *VF* ventricular fibrillation; *ICD* implantable cardioverter defibrillator

### Identification of pathogenic variant

Next generation sequencing was performed on the proband. The average sequencing depths of sample on the targeted regions were 18,992-fold. More than 93.60% targeted regions were covered. We identified a total of 11,583 variants in the proband, including 1232 non-synonymous variants, 1494 synonymous variants, 8857 intronic variants and variants in un-translated regions (UTRs) (Additional file 1: Table S1). After filtering common ones, 82 non-synonymous variants distributed in 42 genes were left. Through screening of SCD associated genes, 4 novel heterozygous non-synonymous variants, including 2 missense variants, 1 non-sense variant and 1 frame-shift variant were selected for further in silico analysis (Table 2). Prediction tools yielded controversy results on A-kinase anchoring protein 9 (*AKAP9*) *c.10714C > G* and filamin C (*FLNC*) *c.7778C > G*, favoring them as harmless polymorphisms, thus their clinical significance was uncertain. Though spectrin repeat containing nuclear envelope protein 1 (*SYNE1) c.25954C > T* non-sense variant was predicted to be disease-causing by MutationTaster, none of the family members presented with neuromuscular disorder as previously reported [15]. The *DSP c.832delG* (Fig. 1a) was predicted to be disease-causing by MutationTaster, PolyPhen-2 and SIFT. Sanger sequencing further revealed that the proband’s father (I-2), her two sisters (III-3 and III-7) and her daughter (IV-1) carried *AKAP9 c.10714C > G.* The proband’s mother (I-3), her youngest sister (III-7) and her niece (IV-4) carried *FLNC c.7778C > G.* The proband’s father (I-2), her youngest sister (III-7), her younger brother (III-5) and her nephew (IV-3) carried *DSP c.832delG* (Table 1). Hence only *DSP c.832delG* was co-segregated with positive phenotype in those characterized members of this family (Table 1; Fig. 1b), supporting the possible pathogenic role of this novel variant. According to ACMG criteria, *AKAP9 c.10714C > G*, *FLNC c.7778C > G* and *SYNE1 c.25954C > T* variants were not co-segregated with positive phenotype in the current family (Table 1), hence, they were classified as benign strong 4 (BS4). However, the *DSP c.832delG* variant, as a frame-shift mutation, was well co-segregated with positive phenotype with in the family, thus was classified as pathogenic very strong (PVS1).

**Table 2:** In silico predictions of 4 novel non-synonymous variants

Gene	cDNA alteration	AA alteration	Effect	Mutation Taster	PolyPhen-2	SIFT
*AKAP9*	*c.10714C > G*	*p.P3572A*	Missense	Polymorphism (0.99)	Benign 0.003	Tolerate (0.86)
*FLNC*	*c.7778C > G*	*p.T2593S*	Missense	Disease causing (0.99)	Benign0.055	Tolerate (0.25)
*SYNE1*	*c.25954C > T*	*p.R8652X*	Nonsense	Disease causing (0.99	Disease causing	Disease causing
*DSP*	*c.832delG*	p.A278Pfs*39	Frame-shift	Disease causing0.99	Disease causing	Disease causing

*DSP* Desmoplakin; *AKAP9* A-kinase anchoring protein 9; *FLNC* filamin C; *SYNE1* spectrin repeat containing nuclear envelope protein 1; *AA* amino acid

### *DSP c.832delG* led to truncated *DSP* mRNA and protein expression, increased *JUP* and decreased β-catenin expression in the nuclear

**Fig. 3:**
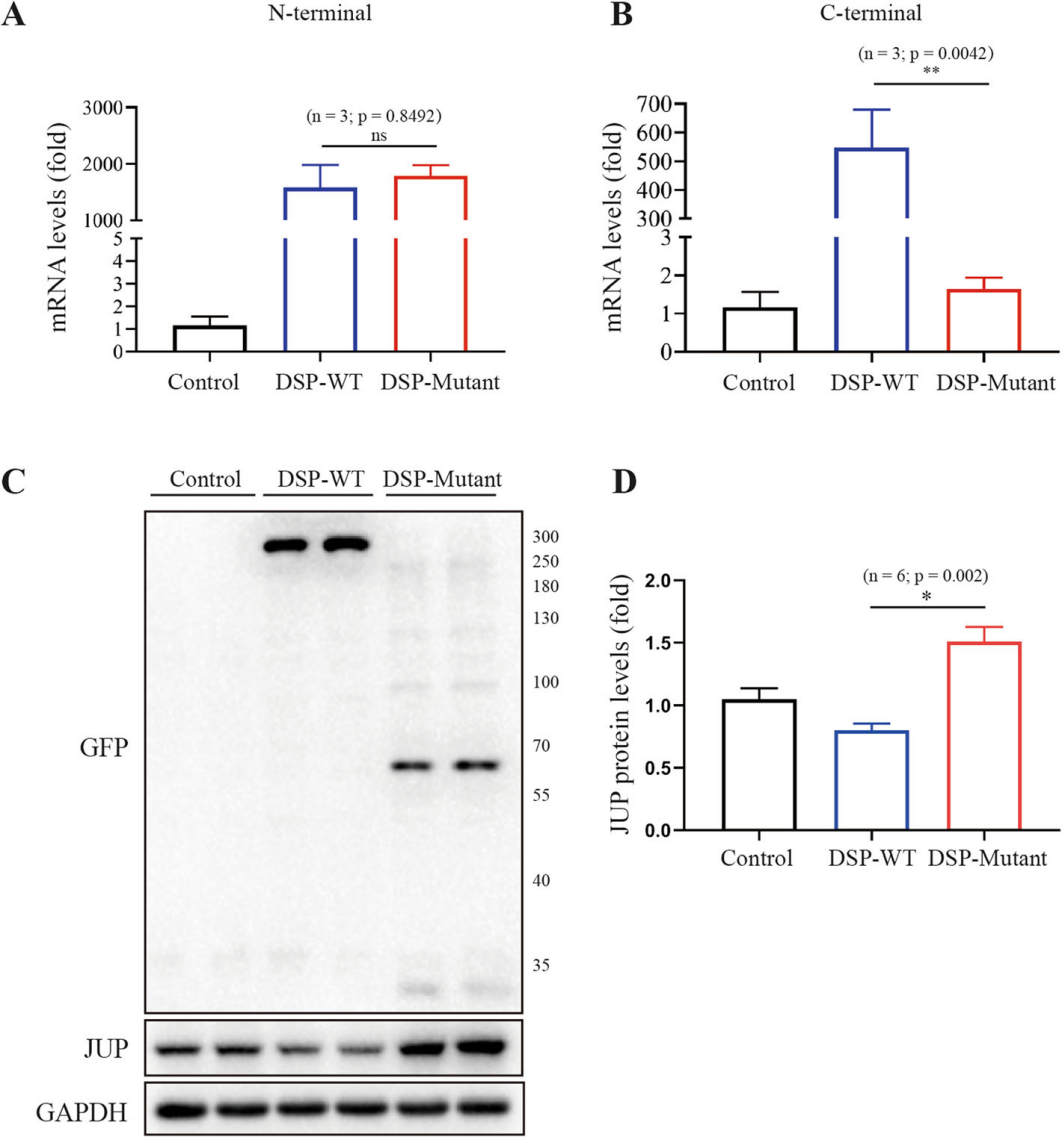
*DSP* mRNA expression, total *DSP* and *JUP* protein expression. HEK293T cells were transfected with either blank, wild type or mutant plasmids. Blank plasmids without *DSP* gene served as control group. **a**-**b** qPCR analysis for *DSP* mRNA levels in the N-terminal and C-terminal of the *c.832delG* mutation site*.* There were no significant differences between mutant and wild-type in mRNA levels in the N-terminal side of *DSP* mutation, whereas, mRNA level in C-terminal side of *DSP* mutation was only elevated in cells transfected with wild type plasmid transfection; **c**-**d***GFP* antibody was used to exam the length of protein expressed in whole cell lysates. Mutant *DSP* protein was much shorter than wild type, suggesting truncation effect of the mutation. *JUP* expression was significantly increased in the mutant group. *GAPDH* served as an internal control. *DSP*, Desmoplakin; *JUP,* Junction plakoglobin; WT, wild type

**Fig. 4:**
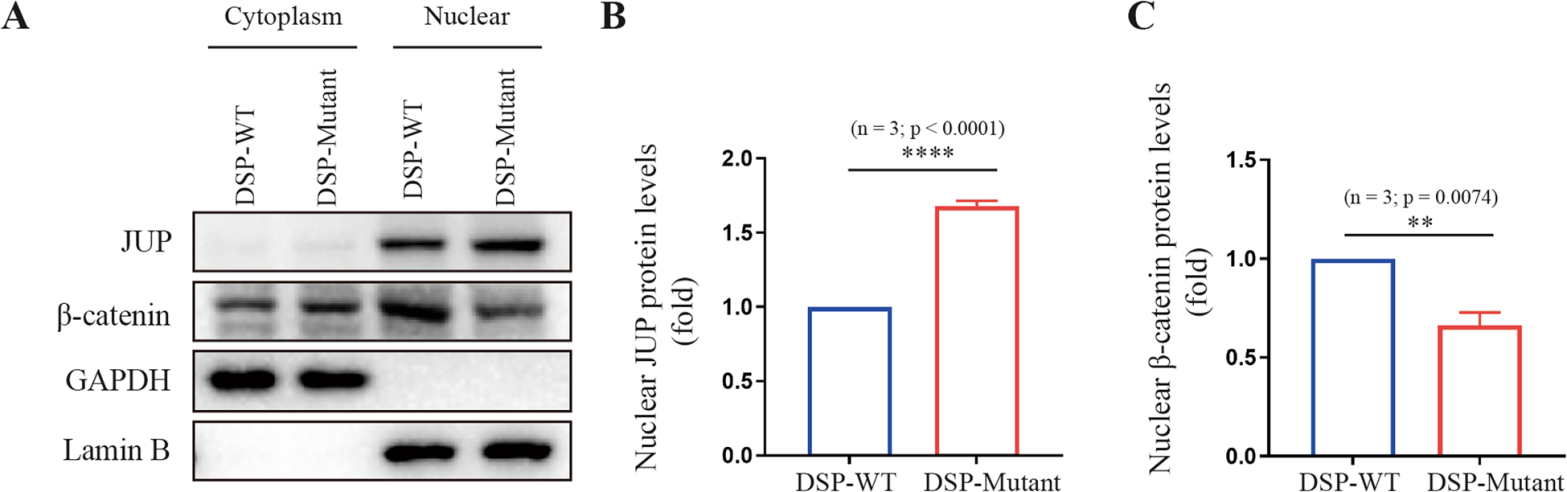
*JUP* and β-catenin expression in cytoplasm and nuclear, separately. HEK293T cells were transfected with either wild type or mutant plasmids. **a**-**b*** JUP* was significantly upregulated and **a**-**c** β-catenin was downregulated in cells transfected with mutant type in the nuclear, rather than cytoplasm, when compared with wild type *DSP*. GAPDH served as an internal control in the cytoplasm and Lamin B served as an internal control in the nuclear. Blank plasmids carrying no *DSP* gene served as control group. *DSP*, Desmoplakin; *JUP*, Junction plakoglobin; *WT*, wild type

**Fig. 5:**
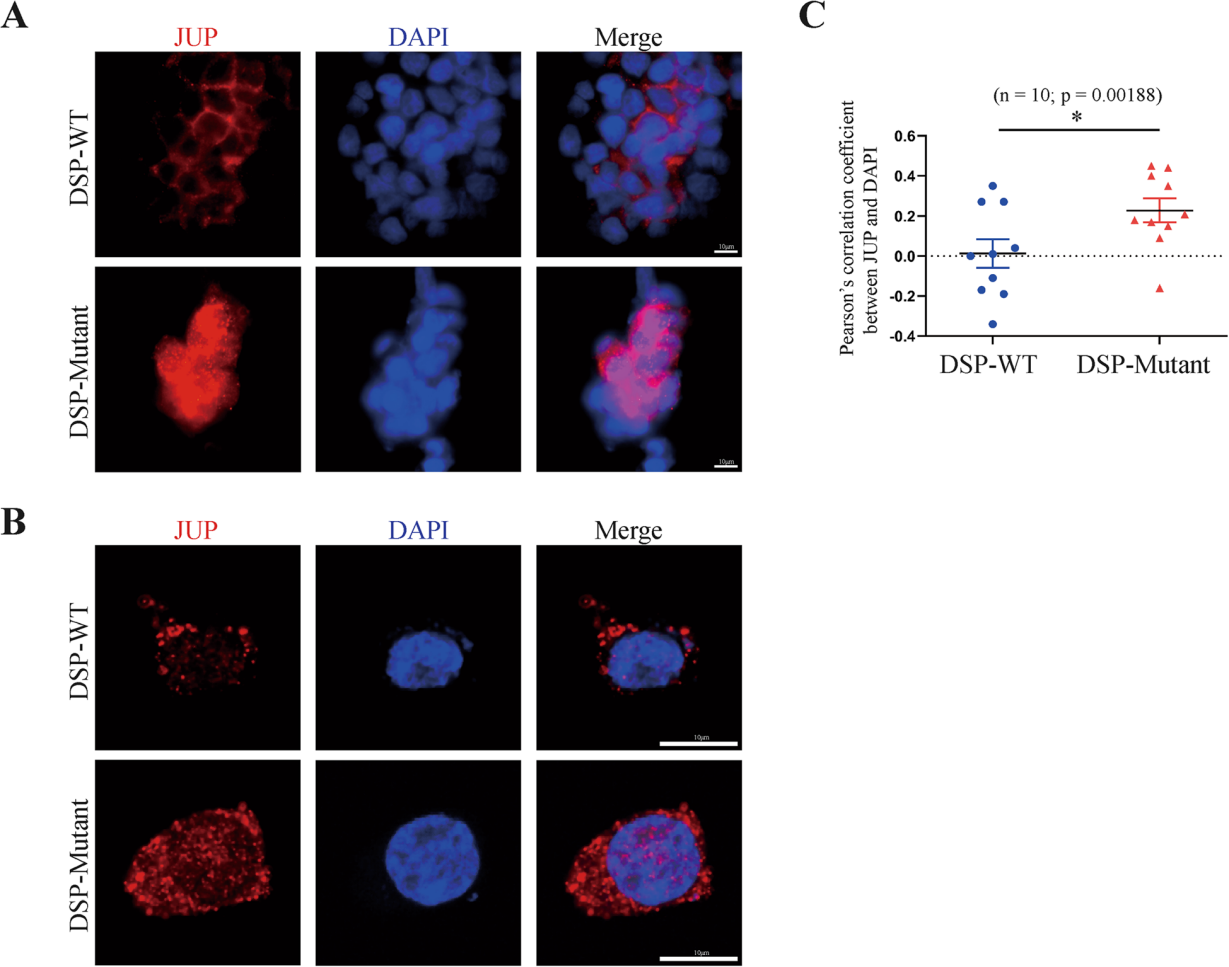
Immunofluorescent staining examined *JUP* expression levels with either wild type or mutant *DSP.* HEK293T cells were transfected with either wild type or mutant plasmids. Blue indicate nuclear (DAPI) and red indicate *JUP*. **a** Representative images of the immunofluorescent staining of transfected HEK293T cells; **b** Confocal microscopic detection of the colocalization of *JUP* with DAPI; **c** Colocalization analysis of *JUP* and DAPI (*n* = 10). DSP, Desmoplakin; JUP, Junction plakoglobin; WT, wild type

The *DSP c.832delG* led to a frame shift and a premature termination codon (p.A278Pfs*39) (Fig. 1c), producing a truncated protein of 315 amino acids, compared with full-length of 2871 amino acids. Real-time PCR found that there were no significant differences between mutant and wild-type in mRNA levels in the N-terminal side of *DSP* mutation, whereas, mRNA levels in C-terminal side of *DSP* mutation were only elevated in wild type cells (Fig. 3a-b), indicating the mRNA translation following the truncation was completely impaired. Western-bolt did not shown a difference of protein expression between wild type and *DSP c.832delG* when using a DSP primary antibody, hence, protein truncation was examined using GFP antibody. Over-expression of plasmids carrying *DSP c.832delG* presented with significantly shortened protein, when compared with wild type (Fig. 3c), suggesting a truncating effect caused by the mutation. We then tested the down-stream protein change separately in cytoplasm and nuclear. *DSP c.832delG* over-expression led to upregulation of *JUP* and downregulation of β-catenin in the nuclear, without affecting their expression in the cytoplasm (Fig. 4a-c), when compared with wild type plasmids. Immunofluorescence through confocal microscopy confirmed the up-regulation of nuclear *JUP* upon transfection of mutant type plasmids (Fig. 5a-c), indicating accumulation of nuclear *JUP* and suppression of Wnt/β-catenin signaling pathway may play a key role in the pathogenesis of AC due to *DSP c.832delG*.

## Discussion

In the current study, through targeted next generation sequencing platform covering a board rang of inherited cardiovascular disease genes, a novel frame-shift variant *DSP c.832delG* is identified in a large SCD family. CMR unveils the typical manifestations of myocardium thinning, fatty replacement and severely impaired heart function, particular in the right heart of the variant carriers, fulfilling the international Task Force criteria for the diagnosis of AC [4]. Functional study on HEK293t cells reveals truncation of *DSP* protein, down-regulation of *JUP* and up-regulation of β-catenin expression in nuclear, but not cytoplasm upon transfection of plasmids with *DSP c.832delG.*

Desmoplakin, a member of the plakin family, anchors other desmosome components to intermediate filaments as to maintain the integrity of desmosome structure [16]. SCD is reported to be more prevalent in *DSP* defect patients, especially truncations [17], when compared with other desmosome defects [9]. In our AC family, 4 family members present with SCD/aborted SCD as first clinical manifestation, and the VT/VF survivor carries *DSP c.832delG* truncation, consistent with previous findings. It has been proposed that *DSP* missense mutation exert a negative dominant effect whereas non-missense mutation exert haploinsufficiency [18], leading to phenotypic discrepancy. *DSP* missense mutation presents with more severe phenotype than non-missense mutation [19], such as earlier disease onset and more prevalence of lethal arrhythmia. However, this correlation is inconsistently reported in clinical studies. Up to date, the largest AC cohort with *DSP* mutation recruiting 27 patients suggests that non-missense mutations is only associated with left-dominant forms [10]. In the current study, despite normal TTE, CMR exam sensitively detects that 2 of our *DSP c.832delG* carriers present mild to moderate left ventricle involvement, nevertheless, right ventricular impairment is dominant, suggesting phenotype is possibly mutation-dependent. Apparently, larger sample of AC cohort with various types of *DSP* mutation will be needed to further explore the genotype-phenotype correlation.

The canonical Wnt/β-catenin signaling is considered to play a central role in the pathogenesis of AC with *DSP* defects [20]. Non-specific heterozygous *DSP*-deficient mice demonstrate substantial adiposity and fibrosis in the ventricular myocardium, recapturing the human AC phenotype [21]. Nuclear translocation of the desmosomal protein plakoglobin (JUP) and suppression of Wnt/β-catenin signaling pathway activity are found to be the underlying mechanism [21]. However, cardiac-restricted *DSP*-deficient mice develop a biventricular form of AC and no significant changes in JUP or β-catenin expression were detected [22], indicating that mechanisms other than Wnt pathway are responsible. In addition, silencing in HL-1 cells result in decreased expression and redistribution of the *Na*_*v*_*1.5* protein and reduced sodium current [23], indicating an orchestra of canonical and non-canonical pathways synergically modulated the disease pathogenesis. Hence, immortal lymphoblastoid cell lines from the *DSP c.832delG* carriers and non-carriers in this family are established as to investigate the molecular pathogenesis. However, in our study no obvious *DSP* expression is detected by either western-blot or flow cytometry (data not shown), hindering the utilization of this cell line in downstream study. Therefore, plasmid carrying *DSP c.832delG* is constructed and transfected into HEK293T cells. Upregulation of *JUP* and downregulation of β-catenin in the nuclear suggest canonical Wnt/β-catenin signaling pathway is likely to play a central role in the development of AC phenotype as previously reported [21]. However, HEK293T cells are unable to simulate the character of cardiomyocyte, hindering further studies on non-canonical pathways and cardiac phenotype.

Various cell models have been established to explore the potential effect of mutations [24]. Buccal mucosa cells from AC patients exhibit redistribution of desmosomes and gap junction protein, similar to those observed in heart [25]. However, in-depth phenotypic and mechanistic studies are not possible due to its distinct cellular features from cardiomyocytes. Patients-specific induced pluripotent stem cells (iPSc) derived cardiomyocytes contain the unique mutations and complete genetic background [26], thus providing us an ideal model to investigate the precise etiology and molecular mechanism. Moreover, the combination of iPSc and latest genome editing technology, such as CRISPR/Cas9, has been succeeded in correcting LQT causal mutations and reversing phenotype [27, 28], promoting it as a promising approach towards precision medicine, and thereby should be introduced in our future study.

### Limitations

In the current study, only HEK293T, a non-cardiac cell line, is utilized. Though human non-myocardial cell lines have been used as a cell model for investigating adhesive junction functions in AC [29], the effects of mutant *DSP* may differ in HEK293T cells from cardiomyocytes. Furthermore, non-cardiac cells are unable to reproduce the phenotype observed in human disease. Human iPSCs derived cardiomyocytes contain the unique genetic background of the patients and features of cardiac cells, hence they are robust tools to perform future studies and explore the mechanistic pathways. Transgenic animals, especially murine genetic knock-ins, are the most powerful and convincing models to investigate human inherited diseases, and also also be considered in the future studies.

## Conclusion

We find the novel *DSP c.832delG* variant, which is likely causal in our AC family. CMR is a powerful alternative approach for the diagnosis of AC with high spatial and temporal resolution, especially in asymptomatic and echocardiogram negative patients. Future studies using patient-specific stem cells or animal models on the impact of the novel mutation, will be warranted to elucidate its pathogenesis of AC.

## Supplementary information

**Additional file 1.** Next generation sequencing results of the propand.

## Data Availability

The datasets used and/or analyzed during the current study are available from the corresponding author on reasonable request.

## Abbreviations

AC: Arrhythmogenic cardiomyopathy
*AKAP9*: A-kinase anchoring protein 9
CMR: Cardiac magnetic resonance
*DSP*: Desmoplakin
ECG: Echocardiogram
*JUP*: Junction plakoglobin
NGS: Next generation sequencing
SCD: Sudden cardiac death
